# AGE, PHYSICAL ACTIVITY AND MITOCHONDRIAL FUNCTION IN THE STUDY OF MUSCLE MOBILITY AND AGING (SOMMA)

**DOI:** 10.1093/geroni/igac059.3088

**Published:** 2022-12-20

**Authors:** Anne Newman, Terri Blackwell, Paul Coen, Yujia Qiao, Frederico Toledo, Ezequiel Zamora, Nancy Glynn

**Affiliations:** University of Pittsburgh, Pittsburgh, Pennsylvania, United States; California Pacific Medical Center, San Francisco, California, United States; AdventHealth, Orlando, Florida, United States; University of Pittsburgh, School of Public Health, Pittsburgh, Pennsylvania, United States; University of Pittsburgh, School of Medicine, Pittsburgh, Pennsylvania, United States; Wake Forest University School of Medicine, Winston Salem, North Carolina, United States; University of Pittsburgh, School of Public Health, Pittsburgh, Pennsylvania, United States

## Abstract

We studied whether lower physical activity (PA) in older adults may partly explain the age-related decline in muscle mitochondrial function in the SOMMA cohort of 879 men and women, aged 70+. PA was measured both subjectively (CHAMPS questionnaire) and objectively (wrist-worn ActiGraph Link) classifying PA levels as sedentary, light, or moderate to vigorous (MVPA, ≥3 METS). Muscle mitochondrial function was assessed ex vivo via respirometry in permeabilized fiber bundles and as in vivo by 31Phosphorus magnetic resonance spectroscopy (31P-MRS) of the maximal rate of muscle ATP regeneration (ATPMAX in the quadriceps after contraction. Of 809 with respirometry or 31P-MRS, mean age was 76.4, 58.3% were women, 12.7% Black, 85.2% White and 2.1% other race/ethnicity. Mean OXPHOS and ATPMAX values were 1.91 pmol/(s*mg) and 0.016 mM/sec lower per 5-year age increment. Men self-reported more MVPA than women, but women recorded more MVPA with actigraphy. For both self-reported and recorded activity, associations with maximal tissue oxidative phosphorylation (OXPHOS) as well as ATPMAX were stronger for MVPA than for sedentary or light activity. After adjustment for age, sex, and clinic site, OXPHOS was lower for each standard deviation) fewer minutes/week of MVPA [reported, SD=408 min/wk: -3.66 pmol/(s*mg) (95% CI -4.93, -2.39); recorded, SD=600 min/wk: -1.93 pmol/(s*mg) (95% CI -3.31, -0.55)]. Similar associations were noted for ATPMAX. MVPA attenuated the association of age with mitochondrial function by 31–50% for both OXPHOS and ATPMAX, suggesting that MVPA could offset the lower mitochondrial function with age or that lower mitochondrial function impedes MVPA at older ages.

